# Supplementary material for: Transparent tissue in solid state for solvent-free and antifade 3D imaging
Source: Nat Commun. 2023 Jun 9;14:3395. doi: 10.1038/s41467-023-39082-4 (PMC10256715; doi:10.1038/s41467-023-39082-4)
Supplement: Supplementary file 1 — Supplementary Information [file 41467_2023_39082_MOESM1_ESM.pdf]

## Supplementary Information

# Transparent tissue in solid state for solvent-free and antifade 3D imaging

Fu-Ting Hsiao, Hung-Jen Chien, Ya-Hsien Chou, Shih-Jung Peng, Mei-Hsin Chung, Tzu-Hui Huang, Li-Wen Lo, Chia-Ning Shen, Hsiu-Pi Chang, Chih-Yuan Lee, Chien-Chia Chen, Yung-Ming Jeng, Yu-Wen Tien, and Shiue-Cheng Tang

Index of Supplementary Figures, Tables, and Movies

Figures

- Supplementary Fig. 1 (related to Fig. 1a, b)
- Supplementary Fig. 2 (related to Fig. 2)
- Supplementary Fig. 3 (related to Fig. 3b-g)
- Supplementary Fig. 4 (related to Fig. 4)
- Supplementary Fig. 5 (related to Fig. 6a-c)
- Supplementary Fig. 6 (related to Methods)
- Supplementary Fig. 7 (related to Methods)

Tables

- Supplementary Table 1 (Related to Methods)
- Supplementary Table 2 (Related to Methods)

Movies

- Supplementary Movie 1 (related to Fig. 1a, b and Methods)
- Supplementary Movie 2 (related to Fig. 4)
- Supplementary Movie 3 (related to Fig. 4 and Supplementary Fig. 4a-i)
- Supplementary Movie 4 (related to Fig. 4 and Supplementary Fig. 4j-o)
- Supplementary Movie 5 (related to Fig. 5b-j)
- Supplementary Movie 6 (related to Fig. 5a-j and Fig. 7)
- Supplementary Movie 7 (related to Fig. 5l)
- Supplementary Movie 8 (related to Fig. 6a-c)
- Supplementary Movie 9 (related to Fig. 6a-c)
- Supplementary Movie 10 (related to Fig. 7)
- Supplementary Movie 11 (related to Fig. 7)
- Supplementary Movie 12 (related to Fig. 7)

## *Supplementary Fig. 1*

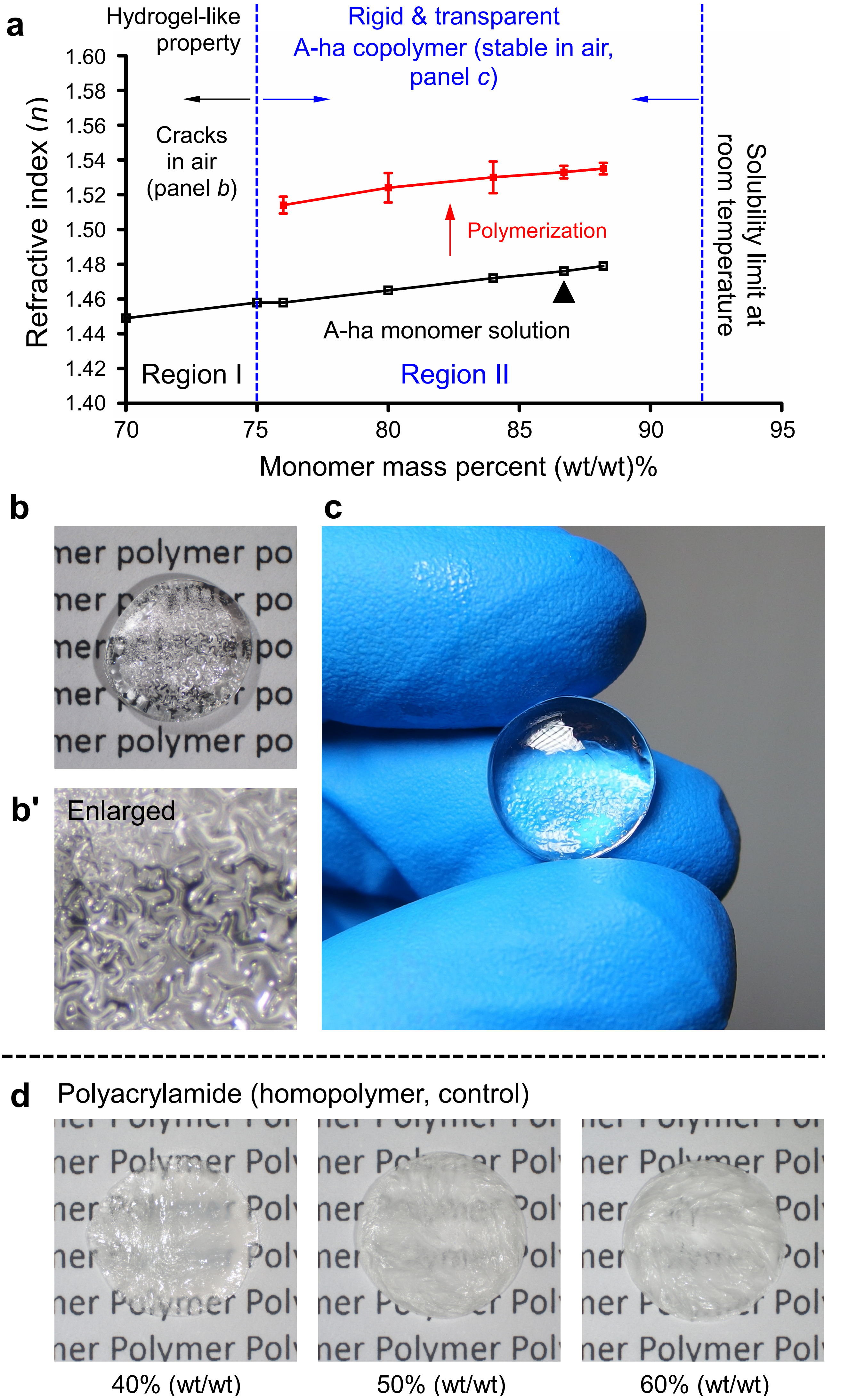


**Supplementary Figure 1** (related to Fig. 1a, b)**. Gel-like vs. rigid acrylamide-based A-ha copolymer.** Acrylamide and n-hydroxymethyl acrylamide (molar ratio 1:1) were polymerized at low (≤75%, Region I) vs. high (≥76%, Region II) mass fraction (**a**). The former developed cracks in air and became opaque (**b** and **b'**; 75% wt/wt), while the latter stayed rigid and transparent in air and can be stored at room temperature without losing its optical (high-$n$) property (**c**; 86.7% wt/wt, $n$=1.53; 24 independent A-ha syntheses). 6 independent A-ha syntheses at 88.2% wt/wt and 4 independent A-ha syntheses at 84%, 80%, and 76% wt/wt were used to estimate the copolymer refractive indexes. Data are presented as means ± standard deviation. Arrowhead in *a* indicates the monomer mass fraction (86.7% wt/wt) used in routine A-ha copolymer synthesis. (**d**) Opaque polyacrylamide homopolymer in air (photo-polymerized at 40%, 50%, and 60% wt/wt). Acrylamide reaches solubility limit at 60% wt/wt in water at room temperature.

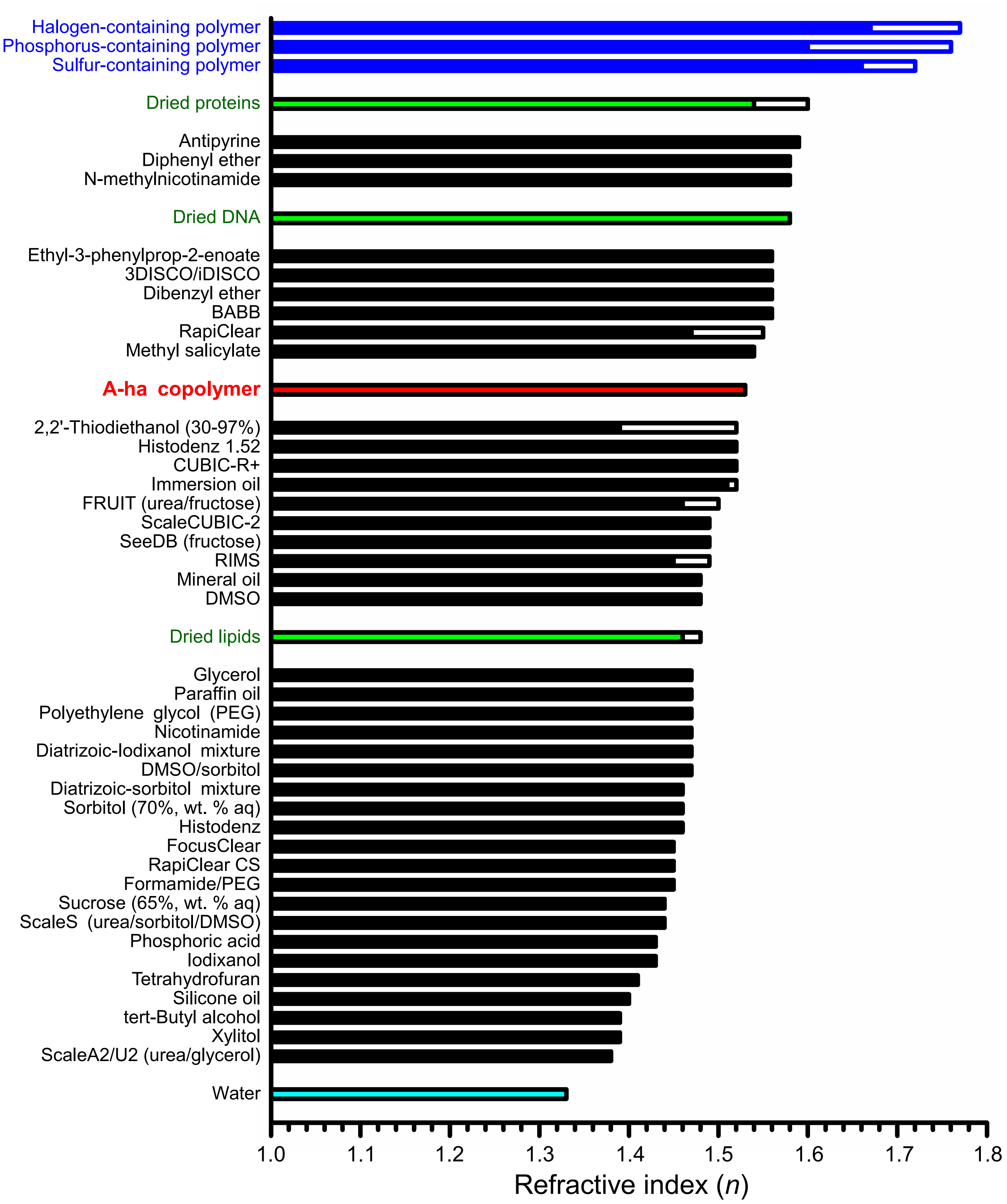


**Supplementary Figure 2** (related to Fig. 2)**. Comparison of refractive indexes among high-*n* polymers, cellular components** (proteins, DNA, and lipids)**, and tissue-clearing reagents.** Empty bar indicates the range of refractive indexes of a polymer, cellular component, or reagent[1-7,17,29-31].

## *Supplementary Fig. 3*

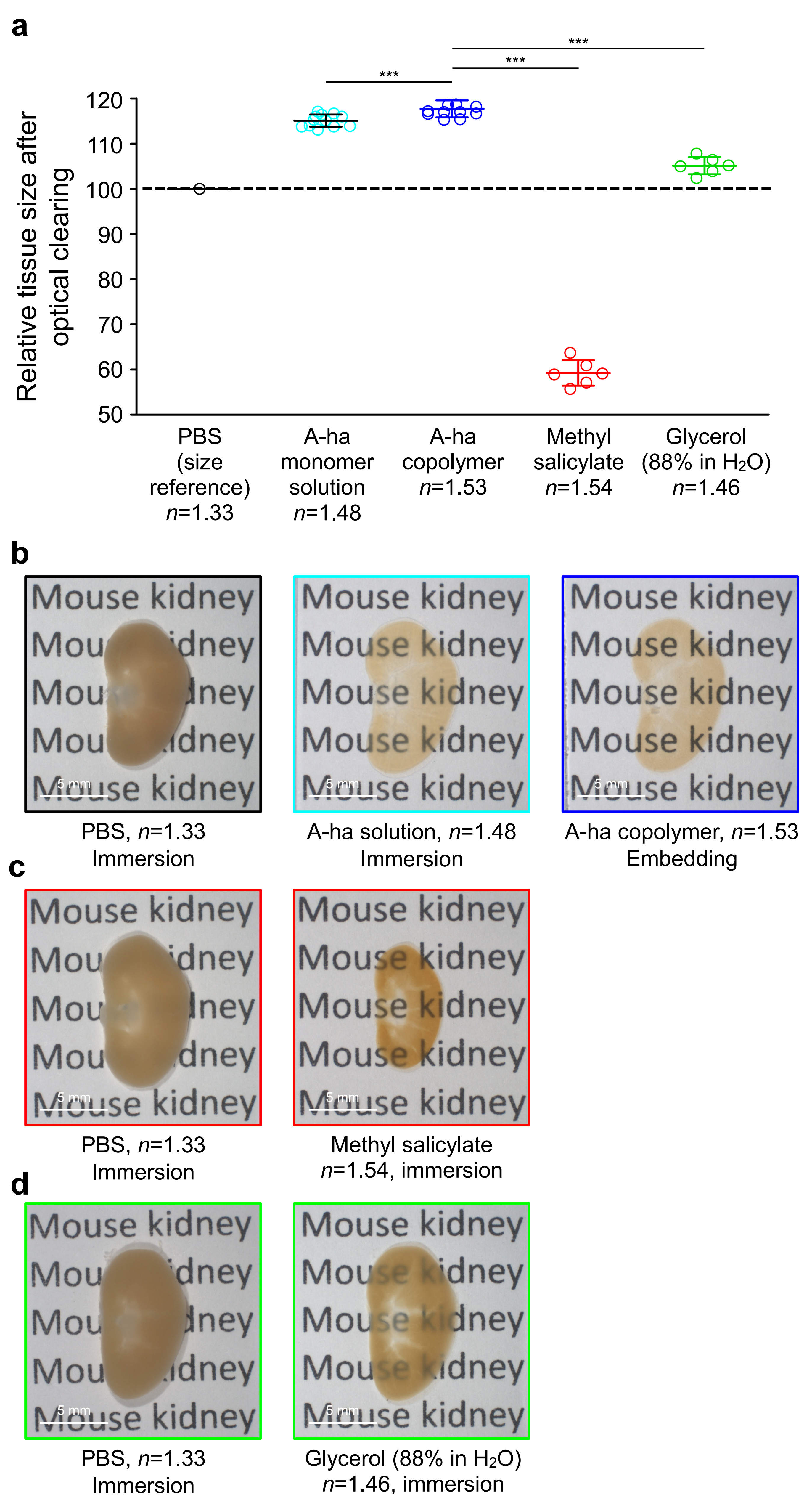


**Supplementary Figure 3** (related to Fig. 3b-g)**. Mild tissue expansion of A-ha-based clearing.** (**a**) Tissue expansion or shrinkage after immersed/embedded in high-*n* media for optical clearing. Pixels of mouse kidney were used to estimate the change. Expressed as % of kidney in PBS. Twelve independent A-ha immersions/embeddings and six independent methyl salicylate and glycerol immersions were performed for the quantitative measurement. ***p<0.001 vs. tissue in A-ha copolymer (two-sided unpaired Student's t-test). Data are presented as means ± standard deviation. (**b-d**) Representative images of A-ha, methyl salicylate, and glycerol-based optical clearing of mouse kidney (500 µm in thickness). **(e)** Quantitative measurement of tissue size via stereomicroscopy. The kidney specimen was first immersed in PBS (between coverslips with a 500-µm spacer) and then imaged via stereomicroscopy (Carl Zeiss, SteREO Discovery.V12) to record the pixels occupied by the tissue (defined as 100). Afterward, the tissue was cleared in the A-ha copolymer (or clearing liquid) and then imaged again under the same microscope. The number of pixels recorded in the clearing condition was divided by that in PBS to estimate the relative tissue size after optical clearing (y axis of panel *a*). In the process, the same background "Mouse kidney" was placed under the specimen to serve as the size standard (relative to tissue) and to demonstrate the change in tissue transparency.

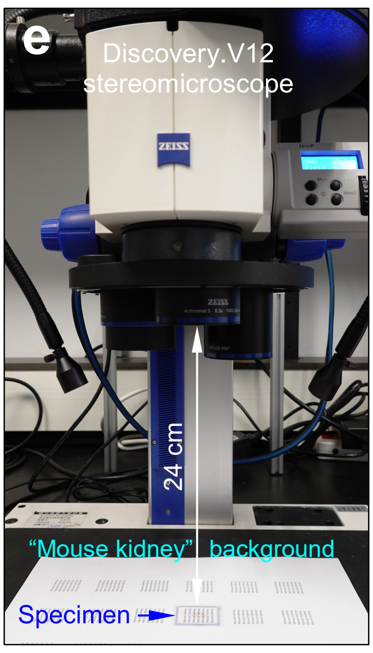

## Mouse brain

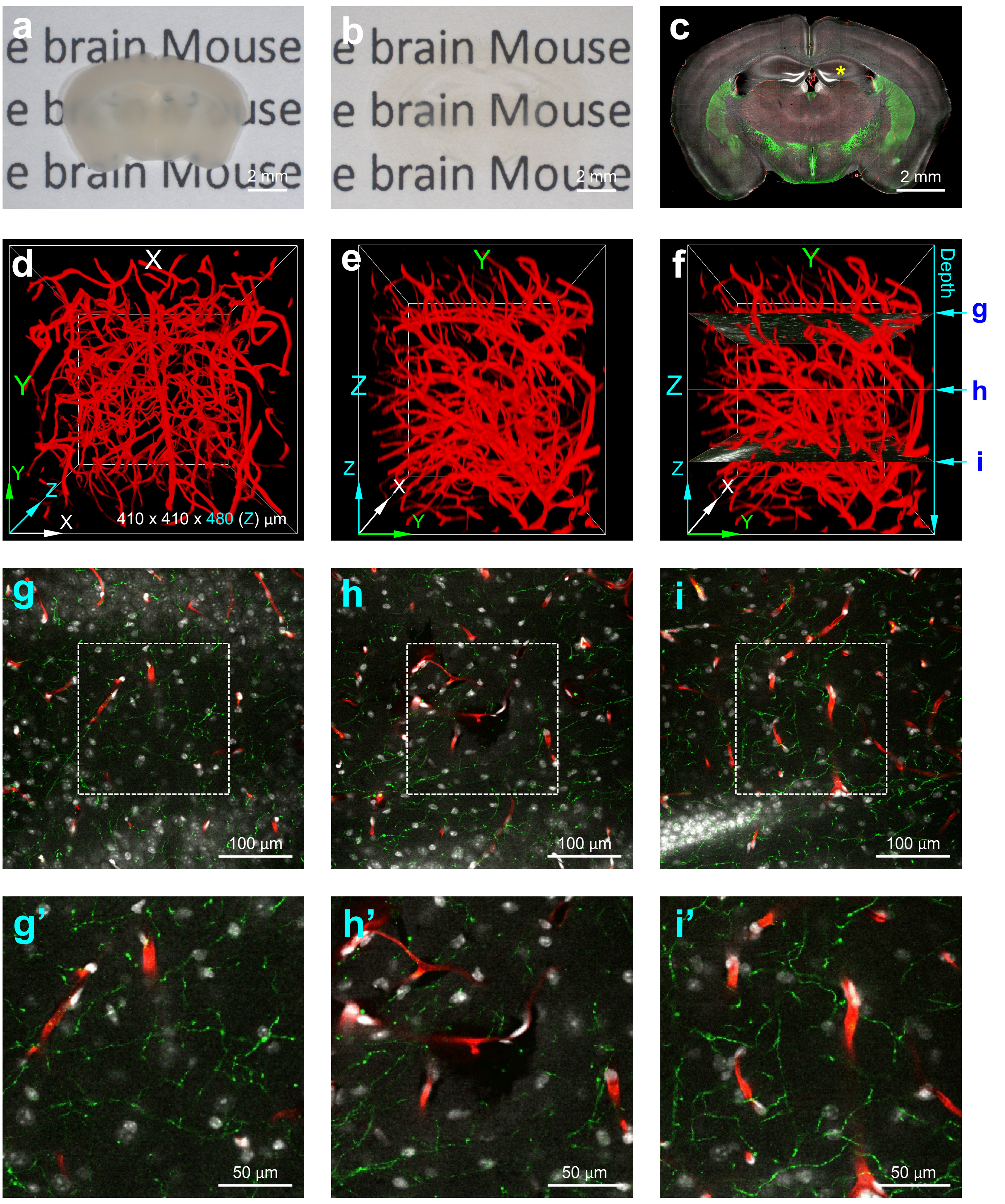


## Mouse kidney

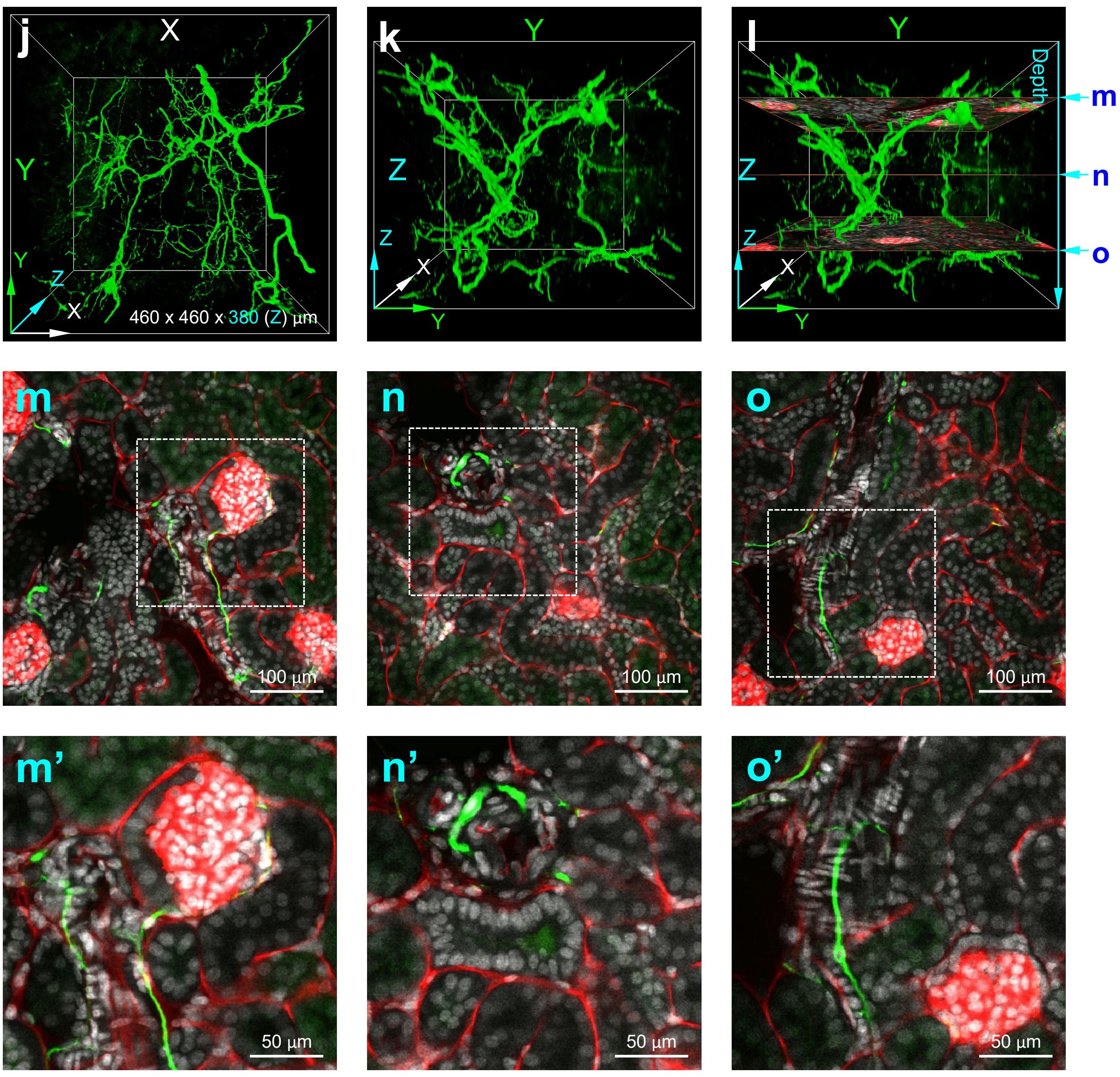


**Supplementary Figure 4** (related to Fig. 4)**. Tissue clearing and in-depth mouse brain and kidney imaging in A-ha copolymer. (a, b)** Opaque vibratome section of mouse brain in PBS (~500 µm in thickness) vs. in A-ha copolymer (transparent). **(c)** Fluorescence tissue map of mouse brain in A-ha copolymer. *a-c* examine the same brain. Green, tyrosine hydroxylase (TH) staining; red, perfusion labeling of blood vessels; white, DAPI. Asterisk indicates the hippocampal area examined in *d-f* (3D projection) and *g-i* (2D image). **(d-f)** 3D vascular projection. *d* and *e* are projections from the XY and YZ planes, respectively. Z, imaging depth. In *f*, depths at 110, 240, and 360 µm along focal depth (Z axis) are labeled and their XY images are presented in *g*, *h*, and *i*, respectively. **(g-i)** In-depth images of $TH^+$ nerve fibers. Boxes in *g-i* are enlarged in *g'-i'*. **(j-o)** 3D projection and 2D image of mouse kidney innervation. The kidney is optically cleared in the A-ha copolymer as presented in **Fig. 3f**. Green, tubulin beta 3 (TUBB3, Biolegend, #657402) staining of nerves; red, perfusion labeling of blood vessels; white, DAPI. *j* and *k* are projections from the XY and YZ planes, respectively. In *l*, depths at 80, 190, and 300 µm along focal depth (Z axis) are labeled and their XY images are presented in *m*, *n*, and *o*, respectively. Boxes in *m-o* are enlarged in *m'*-o*'*. Panel *a-i* and *j-o* are derived from two independent tests of mouse brain and kidney labeling, A-ha embedding, and 3D imaging. **Supplementary Movie 3** and **4** show the in-depth recordings of neurovascular signals in *d-i* (hippocampus, $TH^+$) and *j-o* (innervation of glomeruli, $TUBB3^+$), respectively.

## *Supplementary Fig. 5*

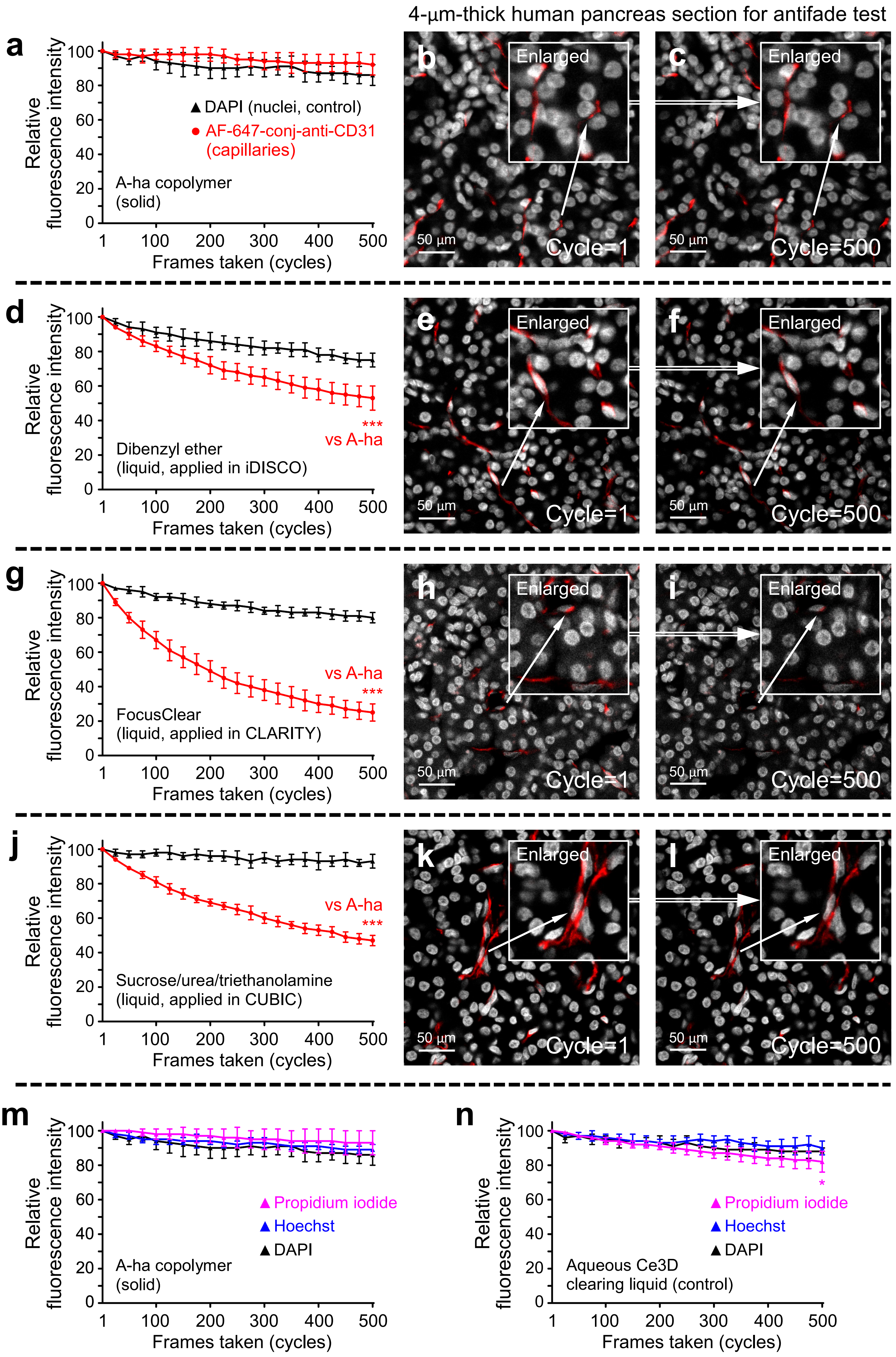


**Supplementary Figure 5** (related to Fig. 6a-c)**.** (**a-l**) Comparison of Alexa Fluor (AF) -647 stability in A-ha (***a-c***) vs. the immersion liquids applied in iDISCO (dibenzyl ether, ***d-f***; organic), CLARITY (FocusClear, ***g-i***; aqueous), and CUBIC (sucrose/urea/triethanolamine solution, ***j-l***; mixture) -based clearing methods. *b-c*, *e-f*, *h-i*, and *k-l*, are representative images. Also see **Supplementary Movie 9** for side-by-side and frame-by-frame comparison of the four conditions. Human pancreas was labeled with DAPI (white, stable control) and anti-CD31 (red, AF-647-conjugated primary antibody; direct immunohistochemistry, indicator). The 500-frame antifade test was performed on the 4-µm microtome section of human pancreas; thus, only the chemical environment, not tissue clearing efficiency, affects the fluorescence detection. On average, the AF-647 signals decreased by 8±6%, 47±7%, 75±5%, and 53±3% in the A-ha copolymer and the solutions of dibenzyl ether, FocusClear, and sucrose/urea/triethanolamine, respectively (n=6 independent antifade tests). Data are expressed as % of fluorescence intensity at cycle=1 (means with standard deviation). ***p<0.001, vs. A-ha (two-sided unpaired Student's t-test). (**m, n**) Stability of nuclear dyes when embedded in A-ha copolymer or immersed in Ce3D clearing liquid. DAPI, Hoechst 33342 (Thermo #H3570), and propidium iodide (PI, Thermo #P21493) show 14±6%, 11±3%, and 7±7% decreases in fluorescence intensity in the A-ha copolymer (4-µm human pancreas section as in ***a-l***), respectively, after 500 frames were taken from the labeled nuclei. In the aqueous Ce3D clearing solution (***n***), the same DAPI, Hoechst, and PI show 12±2% (p=0.42, nonsignificant vs. A-ha), 10±4% (p=0.53, nonsignificant vs. A-ha), and 18±6% (*p<0.05, vs. A-ha) decreases in fluorescence intensity, respectively (n=6 independent antifade tests; two-sided unpaired Student's t-test). Data are presented as means with standard deviation. In the six conditions (***m, n***), the decreases are all less than 20% in the 500-frame antifade test, indicating the intrinsic stability of the three DNA intercalating/binding agents in fluorescence imaging.

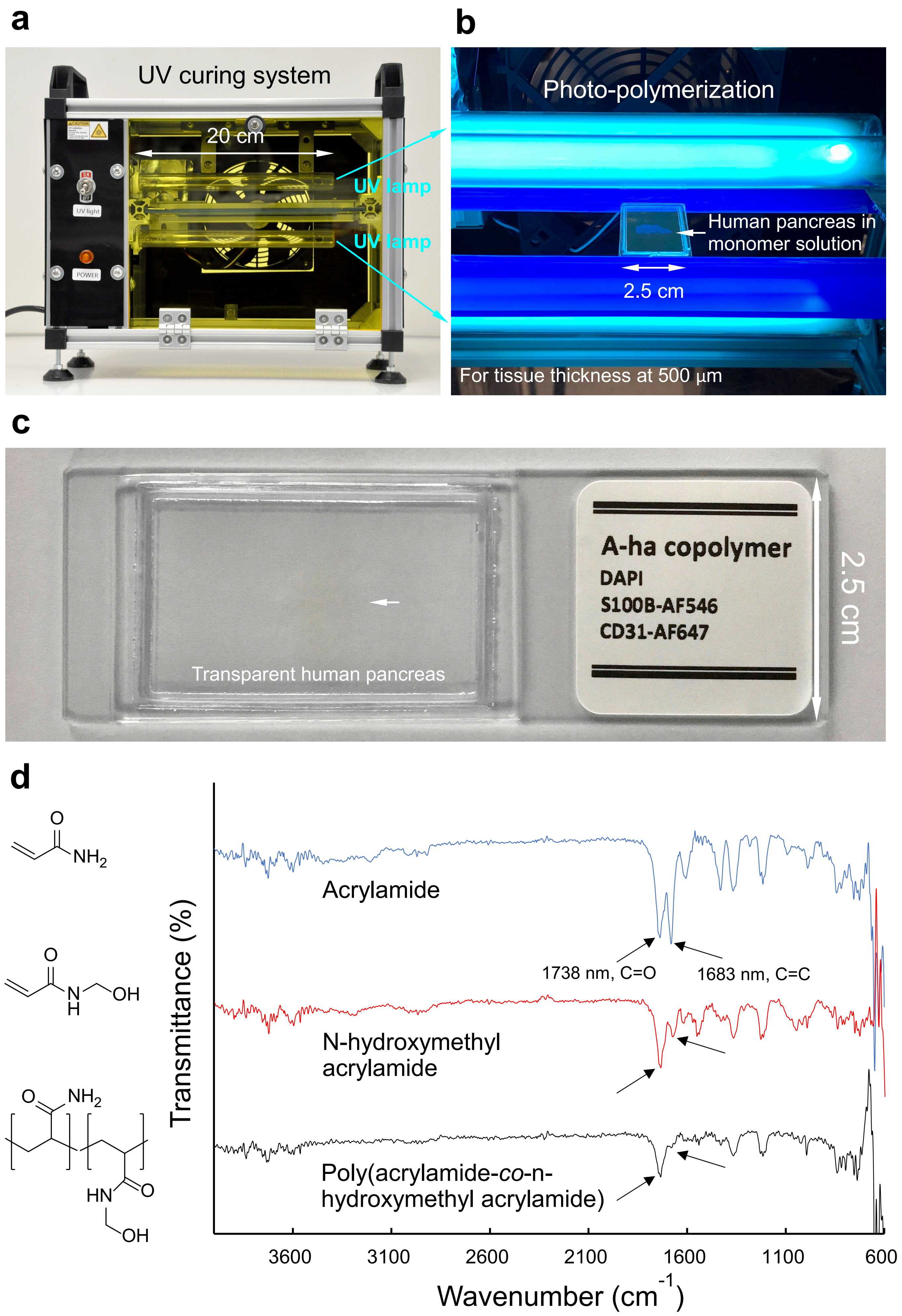


**Supplementary Figure 6** (related to Methods)**. Photo-polymerization and FT-IR analysis of A-ha copolymer. (a, b)** UV curing system and photo-polymerization. Two pairs of UV lamps (Philips TUV PL-L 18W/4P; emission peak at 253.7 nm) were placed at the top and bottom of the specimen for irradiation (irradiance: 9.6 mW/cm$^2$). Tissue (human pancreas) sandwiched between two coverslips via an iSpacer (SunJin Lab) was immersed in the monomer solution with Irgacure 2959 photoinitiator (0.04% mass fraction) to facilitate photo-polymerization. **(c)** Transparent human pancreas in A-ha copolymer on standard microscope slide. The fluorescent labeling is antifade and ready for 3D confocal and/or super-resolution imaging. **(d)** FT-IR spectra of reactants (acrylamide and n-hydroxymethyl acrylamide) and product (A-ha copolymer) following the photo-polymerization. After the reaction, a weak or absent absorption peak at 1683 cm$^{-1}$ (alkenes) indicates that the monomers of acrylamide and n-hydroxymethyl acrylamide were polymerized and changed to poly(**a**crylamide-*co*-n-**h**ydroxymethyl **a**crylamide), the A-ha copolymer.

# *Supplementary Fig. 7*

**a** Acquisition parameters for in-depth Airyscan of human pancreas (40x objective)

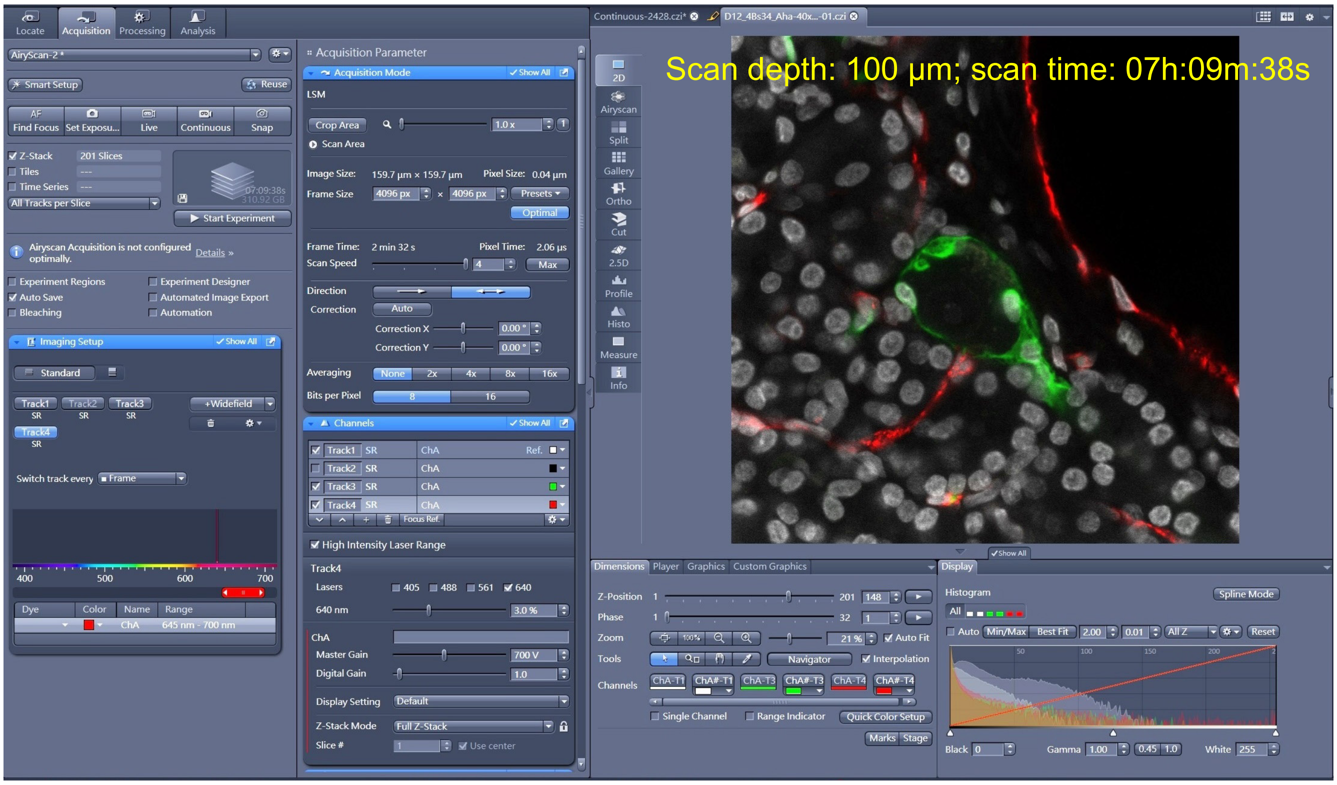


**b** Acquisition parameters for in-depth Airyscan of human pancreas (63x objective)

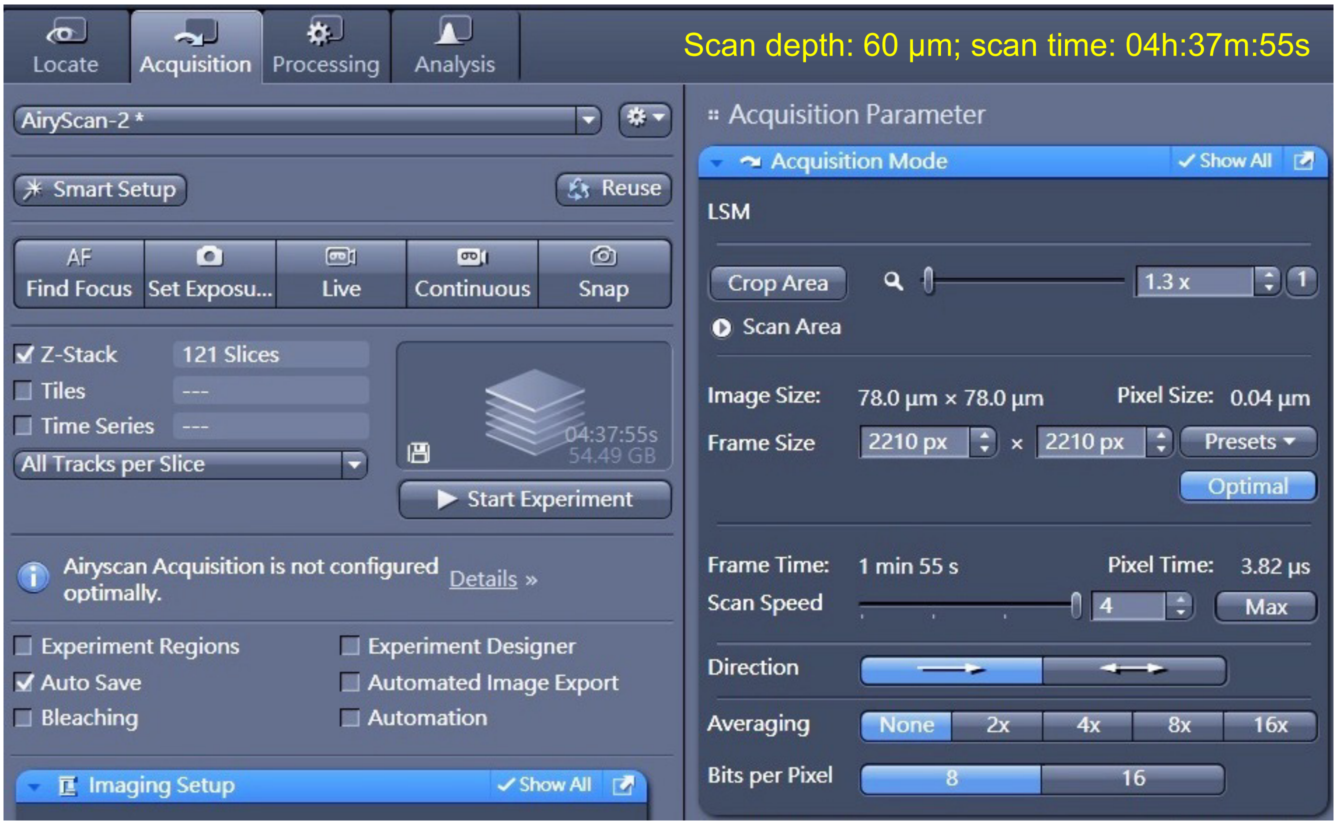


**Supplementary Figure 7** (related to Methods). **Acquisition parameters for in-depth Airyscan of A-ha embedded human pancreas.** Two types of oil-immersion objective, Plan-Apochromat 40x/1.3 Oil DIC (**a**) and Plan-Apochromat 63x/1.4 Oil DIC (**b**) (Carl Zeiss), were sequentially applied to acquire the images presented in **Fig. 7** and **Supplementary Movie 10**.

# *Supplementary Table 1*

**Summary of primary antibodies used in illustrations.**

| Illustration | | Primary antibody (category number) | Dilution | Color code |
|---|---|---|---|---|
| Figure 4 | panel c-j | tyrosine hydroxylase (AB152) | 1: 100 | Green |
| Figure 5 | panel c, e-j | S100B (ab52642) | 1: 100 | Green |
| | | AF-647-conjugated anti-CD31 (ab215912) | 1: 100 | Red |
| Figure 5 | panel k-o | glucagon (ab10988) | 1: 100 | Magenta |
| | | AF-488-cojugated anti-insulin (sc-8033_AF488) | 1: 100 | Blue |
| | | CK7 (ab68459) | 1: 100 | Green |
| Figure 6 | panel a-b, d-e, g-h | S100B (ab52642) | 1: 100 | Green |
| | | AF-647-conjugated anti-CD31 (ab215912) | 1: 100 | Red |
| Figure 7 | panel b-g | S100B (ab52642) | 1: 100 | Green |
| | | AF-647-conjugated anti-CD31 (ab215912) | 1: 100 | Red |
| Suppl. Fig. 4 | panel c-i | tyrosine hydroxylase (AB152) | 1: 100 | Green |
| Suppl. Fig. 4 | panel j-o | tubulin beta 3 (clone AA10, Biolegend, 657402) | 1: 100 | Green |
| Suppl. Fig. 5 | panel a-l | AF-647-conjugated anti-CD31 (ab215912) | 1: 100 | Red |
| Suppl. Movie 2 | | tyrosine hydroxylase (AB152) | 1: 100 | Green |
| Suppl. Movie 3 | | tyrosine hydroxylase (AB152) | 1: 100 | Green |
| Suppl. Movie 4 | | tubulin beta 3 (clone AA10, Biolegend, 657402) | 1: 100 | Green |
| Suppl. Movie 5 | | S100B (ab52642) | 1: 100 | Green |
| | | AF-647-conjugated anti-CD31 (ab215912) | 1: 100 | Red |
| Suppl. Movie 6 | | S100B (ab52642), used in Part 1 | 1: 100 | Green |
| | | AF-647-conjugated anti-CD31 (ab215912) | 1: 100 | Red |
| | | PGP9.5 (ab108986), used in Part 2 | 1: 100 | Green |
| Suppl. Movie 7 | | glucagon (ab10988) | 1: 100 | Magenta |
| | | AF-488-cojugated anti-insulin (sc-8033_AF488) | 1: 100 | Blue |
| | | CK7 (ab68459) | 1: 100 | Green |
| Suppl. Movie 8 | | S100B (ab52642) | 1: 100 | Green |
| | | AF-647-conjugated anti-CD31 (ab215912) | 1: 100 | Red |
| Suppl. Movie 9 | | AF-647-conjugated anti-CD31 (ab215912) | 1: 100 | Red |
| Suppl. Movie 10 | | S100B (ab52642) | 1: 100 | Green |
| | | AF-647-conjugated anti-CD31 (ab215912) | 1: 100 | Red |
| Suppl. Movie 11 | | S100B (ab52642) | 1: 100 | Green |
| | | AF-647-conjugated anti-CD31 (ab215912) | 1: 100 | Red |
| Suppl. Movie 12 | | S100B (ab52642) | 1: 100 | Cyan |
| | | AF-647-conjugated anti-PGP9.5 (ab196173) | 1: 100 | Green |

Note: tyrosine hydroxylase (sympathetic marker), S100B (glial marker), CD31 (endothelial marker), glucagon (islet $\alpha$-cell marker), insulin (islet $\beta$-cell marker), CK7 (epithelial marker), tubulin beta 3 (neuronal marker), and PGP9.5 (neuronal marker).

# *Supplementary Table 2*

**Summary of color codes presented in illustrations.**

| Illustration | | Magenta/Red | Green | Blue/Cyan | White |
|---|---|---|---|---|---|
| Figure 4 | panel a, b (stereomicroscopic image) | | | | |
| | panel c-j | perfusion labeling of blood vessels | TH | | nuclei |
| Figure 5 | panel b (stereomicroscopic image) | | | | |
| | panel c, e-j | CD31 (blood vessels) | S100B | | nuclei |
| | panel d (H&E image) | | | | |
| | panel k-o | glucagon | CK7 | insulin | nuclei |
| Figure 6 | panel a-b, d-e, g-h | CD31 (blood vessels) | S100B | | nuclei |
| Figure 7 | panel a (stereomicroscopic image) | | | | |
| | panel b-g | CD31 (blood vessels) | S100B | | nuclei |
| Suppl. Fig. 4 | panel c-i | perfusion labeling of blood vessels | TH | | nuclei |
| | panel j-o | perfusion labeling of blood vessels | tubulin beta 3 | | nuclei |
| Suppl. Fig. 5 | panel b-c, e-f, h-i, k-l | CD31 (blood vessels) | | | nuclei |
| Suppl. Movie 2 | | perfusion labeling of blood vessels | TH | | nuclei |
| Suppl. Movie 3 | | perfusion labeling of blood vessels | TH | | nuclei |
| Suppl. Movie 4 | | perfusion labeling of blood vessels | tubulin beta 3 | | nuclei |
| Suppl. Movie 5 | (H&E, left; fluorescence, right) | CD31 (blood vessels) | S100B | | nuclei |
| Suppl. Movie 6, part 1 | (H&E, upper left; fluorescence, right) | CD31 (blood vessels) | S100B | | nuclei |
| Suppl. Movie 6, part 2 | (stereomicroscopic image, upper left; fluorescence, right) | CD31 (blood vessels) | PGP9.5 | | nuclei |
| Suppl. Movie 7 | | glucagon | CK7 | insulin | nuclei |
| Suppl. Movie 8 | | CD31 (blood vessels) | S100B | | nuclei |
| Suppl. Movie 9 | | CD31 (blood vessels) | | | nuclei |
| Suppl. Movie 10 | | CD31 (blood vessels) | S100B | | nuclei |
| Suppl. Movie 11 | | CD31 (blood vessels) | S100B | | nuclei |
| Suppl. Movie 12 | | | PGP9.5 | S100B | nuclei |

Note: TH, tyrosine hydroxylase (sympathetic marker), S100B (glial marker), CD31 (endothelial marker), glucagon (islet α-cell marker), insulin (islet β-cell marker), CK7 (epithelial marker), tubulin beta 3 (neuronal marker), and PGP9.5 (neuronal marker).
