# Supplementary material for: Transparent tissue in solid state for solvent-free and antifade 3D imaging
Source: Nat Commun. 2023 Jun 9;14:3395. doi: 10.1038/s41467-023-39082-4 (PMC10256715; doi:10.1038/s41467-023-39082-4)
Supplement: Supplementary file 4 — Description of Additional Supplementary Files [file 41467_2023_39082_MOESM4_ESM.docx]

**Description of Additional Supplementary Files**

**Supplementary Movie 1** (related to Fig. 1a, b and Methods).

**Ultrahigh mass fraction of monomers in solution for photo-polymerization.** Monomers (acrylamide and n-hydroxymethyl acrylamide; molar ratio 1:1) are the predominant component of the solution (86.7% wt/wt; water is only at 13.3% wt/wt). This unique composition leads to the formation of a rigid A-ha copolymer after photo-polymerization (**Supplementary Fig. 1**, vs. soft polyacrylamide gel).

**Supplementary Movie 2** (related to Fig. 4).

**3D fluorescence imaging of mouse liver in A-ha copolymer.** Still images (left panels) show: i) transparent mouse liver in the A-ha copolymer, ii) fluorescent tissue map of the liver, and iii) hepatic nerves following the portal vein in extension. A magnified and in-depth recording of the perivascular innervation (asterisk) is presented in the right panel (arrow). The overlay of transmitted light and fluorescence signals (00:16-00:54) enhances the presentation. Green, sympathetic nerves (tyrosine hydroxylase^+^, immunohistochemistry); magenta, blood vessels (perfusion labeling of endothelium); white, nuclei (DAPI, chemical dye staining). All three types of fluorescent labeling are compatible with the A-ha copolymer embedding for high-resolution 3D microscopy.

**Supplementary Movie 3** (related to Fig. 4 and Supplementary Fig. 4a-i).

**3D neurovascular imaging of mouse brain in A-ha copolymer.** This movie shows an in-depth recording of tyrosine hydroxylase (TH^+^, green) -labeled nerve fibers in the hippocampal area of mouse brain. Red, perfusion labeling of blood vessels. White, nuclei.

**Supplementary Movie 4** (related to Fig. 4 and Supplementary Fig. 4j-o).

**3D neurovascular imaging of mouse kidney in A-ha copolymer.** This movie shows an in-depth recording of tubulin beta 3 (TUBB3^+^, green) -labeled nerve fibers associated with the mouse renal vasculature and glomeruli. Red, perfusion labeling of blood vessels. White, nuclei.

**Supplementary Movie 5** (related to Fig. 5b-j).

**Integration of clinical histology (left) and human pancreas imaging in A-ha copolymer (right).** This movie shows the duct lesion presented in **Fig. 5b-j**. Adjacent microtome (H&E stain, gold standard) and vibratome (fluorescence stain) sections reveal the peri-lesional neurovascular networks. Left: H&E image (confirmation of duct lesion, low-grade PanIN). Right: in-depth glial and vascular imaging. Green, S100B (glia); red, CD31 (blood vessels); white, DAPI (nuclei).

**Supplementary Movie 6** (related to Fig. 5a-j and Fig. 7).

**Multimodal imaging of peri-lesional ganglia in human pancreas.**

**Part 1 (00:00-00:12)** **shows matched H&E and fluorescence images.** Left (still images): lobular and micro-environment of the ganglion (S100B^+^; peri-lesional and peri-lobular). The matched H&E (gold standard) and fluorescence images identify the low-grade PanIN and ganglion. Right (movie): in-depth recording of the ganglion (3 neurons enclosed by glia). Airyscan (32-detector array, super-resolution mode; Carl Zeiss) was used to detect the dimly stained neuronal nuclei, which otherwise cannot be easily visualized with the standard confocal microscopy. Note that DAPI signals were enhanced at the second half (00: 06-00:10) to reveal neurons #2 and #3 (vs. neuron #1, 00:02-00:05). The pancreas was labeled with DAPI (white), anti-S100B (green, standard IHC), and anti-CD31 (red, AF-647-conjugated primary antibody).

**Part 2 (00:13-00:52)** **shows matched stereomicroscopic and fluorescence images.** Left (still images): lobular and micro-environment of the ganglion (PGP9.5^+^; peri-lesional and peri-lobular). Brown color in the lesion domain indicates tissue injury. A ganglion in the fluorescence image is enlarged in the 3D projection (arrow) and magnified in the movie. Right (movie): in-depth recording of the PGP9.5^+^ ganglion (2 neurons). Airyscan was used to detect the dimly stained neuronal nuclei (00:31-00:47; rewind, PGP9.5 off). PGP9.5 is a pan-neuronal marker (Abcam, ab108986; note: endocrine islet cells are also PGP9.5^+^).

**Supplementary Movie 7** (related to Fig. 5l).

**3D imaging of human pancreas duct-islet cell cluster in A-ha copolymer.** This movie shows an in-depth recording of the duct-islet cell cluster identified in the peri-lesional region in the human pancreas (**Fig. 5d, k**). The specimen was embedded and preserved in the A-ha copolymer for high-resolution imaging. Magenta, glucagon (α-cells); blue, insulin (β-cells); green, CK7 (duct cells); white, DAPI (nuclei).

**Supplementary Movie 8** (related to Fig. 6a-c).

**Antifade (time series) test of human pancreas imaging in A-ha copolymer.** Tissues were labeled with DAPI (white), anti-S100B (green, standard IHC), and anti-CD31 (red, AF-647-conjugated primary antibody). 40x objective was used to acquire 500 images from the same 320×320-µm region (~30 µm under tissue surface). 10.8 seconds per frame/one cycle. After 500 cycles (90 minutes), the mean fluorescence signals decreased by 16±4%, 26±6%, and 9±2% (six repeats) of the DAPI, S100B, and CD31 labeling, respectively, vs. signals of the first frame (**Fig. 6c**).

**Supplementary Movie 9** (related to Fig. 6a-c).

**Comparison of AF-647 stability in A-ha vs. the immersion liquids applied in iDISCO (dibenzyl ether), CLARITY (FocusClear), and CUBIC (sucrose/urea/triethanolamine solution) -based clearing methods.** Human pancreas was labeled with DAPI (white) and anti-CD31 (red, AF-647-conjugated primary antibody; direct immunohistochemistry). The four conditions are presented in parallel for side-by-side and frame-by-frame comparison of the signal decay (time series confocal imaging; 500 frames). Note that the test was performed on the 4-µm human pancreas slide; thus, only the chemical environment, not tissue clearing efficiency, affects the fluorescence detection.

**Supplementary Movie 10** (related to Fig. 7).

**3D Airyscan super-resolution imaging of human intrapancreatic ganglion.** Left (still images): lobular and micro-environment of ganglion (S100B^+^; intra-lobular and perivascular). Right (movie): in-depth Airyscan of the ganglion. The pancreas was labeled with DAPI (white), anti-S100B (green, standard IHC), and anti-CD31 (red, AF-647-conjugated primary antibody). The antifade feature of A-ha allows two rounds of in-depth Airyscan with 40x (left, for 3D projection of ganglion) and 63x objectives to reveal the dimly stained neuronal nucleus. DAPI (white) signals are enhanced after 00:21 to reveal the neuronal nucleus.

**Supplementary Movie 11** (related to Fig. 7).

**3D Airyscan and image reconstruction of human intrapancreatic ganglion.** The first part of the movie (00:00-01:08) shows in-depth Airyscan of intrapancreatic ganglion. Four neurons with dimly stained nuclei are revealed (00:22-00:42). The pancreas was labeled with DAPI (white), anti-S100B (green, standard IHC), and anti-CD31 (red, AF-647-conjugated primary antibody). Part 2 (01:09-01:52) shows 360-degree projection of the S100B-labeled ganglion. Dimensions of the image stack: 160 (x) × 160 (y) × 130 (z, depth) µm.

**Supplementary Movie 12** (related to Fig. 7).

**3D Airyscan of human intrapancreatic ganglion with paired glial and neuronal labeling.** Left (still images): lobular and micro-environment of ganglion. Right (movie): in-depth Airyscan of the ganglion. The pancreas was labeled with DAPI (white), anti-S100B (cyan, standard IHC), and anti-PGP9.5 (green, AF-647-conjugated primary antibody; Abcam ab196173). The result confirms the A-ha-based 3D super-resolution imaging with direct immunohistochemistry (anti-PGP9.5-AF-647).
